# Supplementary material for: MetaRibo-Seq measures translation in microbiomes
Source: Nat Commun. 2020 Jun 29;11:3268. doi: 10.1038/s41467-020-17081-z (PMC7324362; doi:10.1038/s41467-020-17081-z)
Supplement: Supplementary file 10 — Supplementary Data 7 [file 41467_2020_17081_MOESM10_ESM.zip › File2/Confidence_High_Taxonomy/155185_out.krona.html]

Javascript must be enabled to view this page.

members
magnitude
magnitudeUnassigned
count
unassigned
taxon
rank

155185\_out

301

superkingdom
301
2

976
phylum
301

200643
class
301

301
order
171549

1
family
1853231

1
genus
574697

1
species

SRS049959\_contig\_number\_contig-100\_128.246902
544645

815
family
299

genus
299
10

SRS013638\_contig\_number\_contig-100\_518.128252SRS024492\_contig\_number\_269SRS045713\_contig\_number\_20479SRS045739\_contig\_number\_4885SRS049712\_contig\_number\_18294SRS063489\_contig\_number\_4843SRS1041133\_contig\_number\_18818SRS104311\_contig\_number\_42898SRS893279\_contig\_number\_15271SRS893300\_contig\_number\_10554
816

371601

SRS016954\_contig\_number\_27585SRS017745\_contig\_number\_585SRS047433\_contig\_number\_24366SRS049896\_contig\_number\_23201SRS148424\_contig\_number\_contig-100\_5530.147989
5
species


SRS016989\_contig\_number\_9687SRS893382\_contig\_number\_1600
47678
2
species

1796613

SRS893327\_contig\_number\_3173
1
species

1
species

SRS022071\_contig\_number\_25337
1262736


SRS012849\_contig\_number\_2800SRS014412\_contig\_number\_19021SRS015217\_contig\_number\_17366SRS016056\_contig\_number\_7985SRS019787\_contig\_number\_3649SRS049773\_contig\_number\_26384SRS050801\_contig\_number\_3774SRS051031\_contig\_number\_34721SRS056537\_contig\_number\_43449SRS062654\_contig\_number\_12982SRS1041112\_contig\_number\_5238SRS1041142\_contig\_number\_1233SRS1055022\_contig\_number\_4586SRS1055038\_contig\_number\_1613SRS142980\_contig\_number\_6766SRS143991\_contig\_number\_1593SRS147614\_contig\_number\_10490SRS147977\_contig\_number\_10426SRS148970\_contig\_number\_3091SRS893292\_contig\_number\_9624
338188
species
20

species
2
2292956

SRS018049\_contig\_number\_681SRS052078\_contig\_number\_1907


SRS014736\_contig\_number\_8041SRS018575\_contig\_number\_6146SRS020508\_contig\_number\_1006SRS023583\_contig\_number\_2794SRS045826\_contig\_number\_2706SRS046810\_contig\_number\_contig-100\_10.11SRS048870\_contig\_number\_contig-100\_1882.129759SRS052576\_contig\_number\_contig-100\_44.15553SRS054905\_contig\_number\_15734SRS054956\_contig\_number\_16229SRS056273\_contig\_number\_3106SRS056519\_contig\_number\_2109SRS063928\_contig\_number\_251SRS064557\_contig\_number\_4984SRS065397\_contig\_number\_15231SRS075078\_contig\_number\_24213SRS098717\_contig\_number\_contig-100\_1938.140562SRS101376\_contig\_number\_22265SRS1041134\_contig\_number\_2774SRS1041138\_contig\_number\_12324SRS1041141\_contig\_number\_10037SRS104485\_contig\_number\_429SRS142923\_contig\_number\_19990SRS143523\_contig\_number\_18048SRS145308\_contig\_number\_4443SRS147919\_contig\_number\_12301SRS148424\_contig\_number\_contig-100\_1431.170022SRS892882\_contig\_number\_15279SRS893373\_contig\_number\_15619
1739517
species
29

4
species
162156

SRS016335\_contig\_number\_contig-100\_2239.239760SRS022137\_contig\_number\_contig-100\_1516.129889SRS145497\_contig\_number\_5486SRS147022\_contig\_number\_3896

2
species

SRS011302\_contig\_number\_10307SRS148091\_contig\_number\_3307
821

5
species
556259

SRS015578\_contig\_number\_25082SRS018133\_contig\_number\_12943SRS045244\_contig\_number\_4845SRS098655\_contig\_number\_13555SRS146812\_contig\_number\_44542

183
species
28116

SRS011084\_contig\_number\_24914SRS012273\_contig\_number\_45142SRS012902\_contig\_number\_1379SRS012969\_contig\_number\_34879SRS013098\_contig\_number\_41921SRS013215\_contig\_number\_463SRS013382\_contig\_number\_617SRS013476\_contig\_number\_32240SRS013521\_contig\_number\_13379SRS013687\_contig\_number\_4712SRS014459\_contig\_number\_38008SRS014613\_contig\_number\_1725SRS014683\_contig\_number\_17549SRS014855\_contig\_number\_17769SRS014979\_contig\_number\_35329SRS015065\_contig\_number\_38394SRS015264\_contig\_number\_13473SRS015369\_contig\_number\_6706SRS015579\_contig\_number\_26145SRS015694\_contig\_number\_14395SRS015782\_contig\_number\_44761SRS015854\_contig\_number\_12284SRS015890\_contig\_number\_12219SRS015960\_contig\_number\_1801SRS016018\_contig\_number\_contig-100\_946.96133SRS016095\_contig\_number\_27439SRS016132\_contig\_number\_2155SRS016203\_contig\_number\_12747SRS016381\_contig\_number\_2079SRS016840\_contig\_number\_2744SRS016897\_contig\_number\_1122SRS017103\_contig\_number\_37622SRS017191\_contig\_number\_2316SRS017247\_contig\_number\_9524SRS017433\_contig\_number\_9657SRS017622\_contig\_number\_65SRS017701\_contig\_number\_3535SRS017821\_contig\_number\_3788SRS017859\_contig\_number\_6031SRS018133\_contig\_number\_3231SRS018313\_contig\_number\_7676SRS018427\_contig\_number\_26105SRS018623\_contig\_number\_6530SRS018817\_contig\_number\_17905SRS018936\_contig\_number\_10274SRS018984\_contig\_number\_29414SRS019030\_contig\_number\_contig-100\_1175.104132SRS019068\_contig\_number\_65735SRS019178\_contig\_number\_3469SRS019381\_contig\_number\_13282SRS019381\_contig\_number\_19198SRS019397\_contig\_number\_4414SRS019445\_contig\_number\_21382SRS019693\_contig\_number\_748SRS019910\_contig\_number\_17212SRS019968\_contig\_number\_9692SRS020271\_contig\_number\_contig-100\_103.30487SRS020328\_contig\_number\_16226SRS020622\_contig\_number\_749SRS021219\_contig\_number\_9844SRS021484\_contig\_number\_17059SRS022524\_contig\_number\_10103SRS022713\_contig\_number\_2241SRS023346\_contig\_number\_2651SRS023715\_contig\_number\_23984SRS023829\_contig\_number\_1231SRS023914\_contig\_number\_contig-100\_1227.161573SRS024132\_contig\_number\_14435SRS024331\_contig\_number\_33624SRS024388\_contig\_number\_contig-100\_2706.92808SRS024435\_contig\_number\_3960SRS024549\_contig\_number\_4766SRS024663\_contig\_number\_12729SRS043001\_contig\_number\_7135SRS043701\_contig\_number\_10342SRS044535\_contig\_number\_contig-100\_1302.138553SRS045004\_contig\_number\_27977SRS045645\_contig\_number\_33001SRS046369\_contig\_number\_16293SRS046717\_contig\_number\_5658SRS049995\_contig\_number\_30973SRS050026\_contig\_number\_12094SRS050299\_contig\_number\_1031SRS050422\_contig\_number\_26628SRS050752\_contig\_number\_29079SRS050941\_contig\_number\_21654SRS052697\_contig\_number\_24885SRS053398\_contig\_number\_20304SRS053573\_contig\_number\_977SRS054352\_contig\_number\_contig-100\_214.138998SRS056259\_contig\_number\_1597SRS057478\_contig\_number\_1931SRS057480\_contig\_number\_contig-100\_124.68226SRS057717\_contig\_number\_555SRS058070\_contig\_number\_9068SRS058268\_contig\_number\_211SRS058770\_contig\_number\_2294SRS063518\_contig\_number\_21848SRS063813\_contig\_number\_contig-100\_705.57936SRS064232\_contig\_number\_3743SRS065176\_contig\_number\_16045SRS065504\_contig\_number\_22992SRS074964\_contig\_number\_5733SRS075398\_contig\_number\_2607SRS075821\_contig\_number\_23995SRS075984\_contig\_number\_1936SRS076756\_contig\_number\_19874SRS076976\_contig\_number\_contig-100\_1487.118404SRS077024\_contig\_number\_10112SRS077086\_contig\_number\_1021SRS077194\_contig\_number\_26316SRS077231\_contig\_number\_26084SRS077454\_contig\_number\_contig-100\_1222.117200SRS077502\_contig\_number\_13509SRS077552\_contig\_number\_20002SRS077849\_contig\_number\_28205SRS078242\_contig\_number\_31846SRS078419\_contig\_number\_21619SRS097889\_contig\_number\_16948SRS097958\_contig\_number\_19715SRS098061\_contig\_number\_1428SRS098571\_contig\_number\_33870SRS098881\_contig\_number\_4134SRS101433\_contig\_number\_12352SRS103987\_contig\_number\_25093SRS104084\_contig\_number\_10869SRS1041037\_contig\_number\_9840SRS1041038\_contig\_number\_contig-100\_14.36374SRS1041092\_contig\_number\_9828SRS1041095\_contig\_number\_1810SRS1041136\_contig\_number\_11051SRS1041147\_contig\_number\_4894SRS1041164\_contig\_number\_578SRS104327\_contig\_number\_3306SRS104400\_contig\_number\_12212SRS104975\_contig\_number\_21353SRS1055034\_contig\_number\_12890SRS1055076\_contig\_number\_contig-100\_1525.90814SRS1055095\_contig\_number\_822SRS140513\_contig\_number\_7473SRS140645\_contig\_number\_171SRS142503\_contig\_number\_39475SRS142542\_contig\_number\_5387SRS143085\_contig\_number\_1464SRS143372\_contig\_number\_7292SRS143417\_contig\_number\_1633SRS143466\_contig\_number\_contig-100\_17.180741SRS143598\_contig\_number\_21055SRS143722\_contig\_number\_1071SRS143780\_contig\_number\_1342SRS143876\_contig\_number\_32150SRS143895\_contig\_number\_44467SRS144183\_contig\_number\_39941SRS144297\_contig\_number\_8673SRS144362\_contig\_number\_26959SRS144537\_contig\_number\_5953SRS144603\_contig\_number\_4771SRS146764\_contig\_number\_25656SRS147039\_contig\_number\_3094SRS147088\_contig\_number\_3677SRS147139\_contig\_number\_40750SRS147346\_contig\_number\_49581SRS147425\_contig\_number\_9684SRS147653\_contig\_number\_3022SRS148159\_contig\_number\_46294SRS148196\_contig\_number\_33213SRS148319\_contig\_number\_11886SRS148511\_contig\_number\_14467SRS148817\_contig\_number\_5617SRS149181\_contig\_number\_contig-100\_777.175451SRS149244\_contig\_number\_9264SRS149784\_contig\_number\_27082SRS150029\_contig\_number\_2038SRS893170\_contig\_number\_10118SRS893253\_contig\_number\_191SRS893256\_contig\_number\_1270SRS893285\_contig\_number\_contig-100\_1407.16991SRS893341\_contig\_number\_9703SRS893342\_contig\_number\_2859SRS893358\_contig\_number\_7436SRS893369\_contig\_number\_10517SRS893378\_contig\_number\_19806SRS971276\_contig\_number\_24481


SRS013951\_contig\_number\_37247SRS063190\_contig\_number\_contig-100\_954.94715
469590
2
species

species
2

SRS015431\_contig\_number\_54367SRS019601\_contig\_number\_5561
246787

46506

SRS074670\_contig\_number\_8297SRS148721\_contig\_number\_15721SRS893259\_contig\_number\_4777
species
3

species
3

SRS014287\_contig\_number\_10746SRS019397\_contig\_number\_9619SRS062427\_contig\_number\_2494
2292957


SRS013940\_contig\_number\_14805SRS014923\_contig\_number\_contig-100\_498.246975SRS016267\_contig\_number\_contig-100\_1189.88198SRS020394\_contig\_number\_18068SRS023526\_contig\_number\_9455SRS046068\_contig\_number\_4833SRS048981\_contig\_number\_7357SRS050925\_contig\_number\_18530SRS051882\_contig\_number\_18912SRS062701\_contig\_number\_7060SRS064276\_contig\_number\_27141SRS075021\_contig\_number\_contig-100\_1733.54981SRS075773\_contig\_number\_38381SRS076804\_contig\_number\_20383SRS077127\_contig\_number\_contig-100\_1338.117284SRS097920\_contig\_number\_contig-100\_2128.182926SRS098644\_contig\_number\_24140SRS1041137\_contig\_number\_5378SRS105153\_contig\_number\_14673SRS143148\_contig\_number\_12366SRS148784\_contig\_number\_contig-100\_1882.158410SRS149075\_contig\_number\_14305SRS893288\_contig\_number\_168
818
species
23

2
species

SRS015794\_contig\_number\_contig-100\_2629.79171SRS052027\_contig\_number\_20638
2302926

1
family
2005525

375288
genus
1


SRS020869\_contig\_number\_10838
2292242
species
1
